# Arsenic stress after the Proterozoic glaciations

**DOI:** 10.1038/srep17789

**Published:** 2015-12-04

**Authors:** Ernest Chi Fru, Emma Arvestål, Nolwenn Callac, Abderrazak El Albani, Stephanos Kilias, Ariadne Argyraki, Martin Jakobsson

**Affiliations:** 1 Stockholm University, Department of Geological Sciences and Bolin Centre for Climate Research, SE-106 91, Stockholm, Sweden; 2 Nordic Centre for Earth Evolution, Swedish Museum of Natural History, Department of Paleobiology Box 50007, SE-104 05, Stockholm, Sweden; 3 Uppsala University, Department of Earth Sciences, Paleobiology, SE-752 36, Uppsala, Sweden; 4 Université de Poitiers UMR 7285-CNRS, Institut de Chimie des Milieux et Matériaux de Poitiers-5, rue Albert Turpin (Bât B35) 86073 Poitiers cedex; 5 Department of Economic Geology and Geochemistry Faculty of Geology and Geoenvironment, University of Athens Panepistimiopolis Zographou 157 84 Athens, Greece

## Abstract

Protection against arsenic damage in organisms positioned deep in the tree of life points to early evolutionary sensitization. Here, marine sedimentary records reveal a Proterozoic arsenic concentration patterned to glacial-interglacial ages. The low glacial and high interglacial sedimentary arsenic concentrations, suggest deteriorating habitable marine conditions may have coincided with atmospheric oxygen decline after ~2.1 billion years ago. A similar intensification of near continental margin sedimentary arsenic levels after the Cryogenian glaciations is also associated with amplified continental weathering. However, interpreted atmospheric oxygen increase at this time, suggests that the marine biosphere had widely adapted to the reorganization of global marine elemental cycles by glaciations. Such a glacially induced biogeochemical bridge would have produced physiologically robust communities that enabled increased oxygenation of the ocean-atmosphere system and the radiation of the complex Ediacaran-Cambrian life.

The suggested transition from submarine to widespread subaerial volcanism (refs 1, 2, 3) and continental growth during the Great Oxidation Event (GOE), ~2.45–2.1 billion years ago (Ga), is believed to have triggered increased chemical weathering of trace elements from land to ocean^4^,^5^,^6^,^7^,^8^,^9^,^10^,^11^. This is often correlated to biological stimulation near ocean margins^4^,^5^,^6^,^7^,^8^,^10^,^11^. However, life had to develop strategies to combat the sudden bioavailability of a range of toxic, redox-sensitive elements that became widely accessible because of the GOE. For example, the conservative behavior of As in the presence of oxygen^12^,^13^,^14^ suggests arsenate (As(V)) became globally bioavailable in the oxygen-rich layer enveloping the ocean surface because of global-scale oxidation of arsenite (As(III)). A widely accepted view is that the GOE brought intense weathering of continental sulfide-rich minerals and the amplification of the marine sulfate reservoir as a conseqence^1^,^4^,^9^,^10^,^11^. The oxidation of sulfide-rich rocks is linked to acid rock drainage during the GOE^9^. This process, which arises from the oxidation of pyrite, arsenopyrite and various As sulfides (e.g., orpiment and realgar), is one recognized cause of severe groundwater contamination by As in the modern oxidized environment^12^,^13^,^14^. For example the concentration of As in pyrite alone may be up to 5600 mg kg^−1^ (refs 12,13). Moreover, As-rich pyrite is more susceptible to chemical weathering than pure pyrite^13^. Thus weathering fluxes of As-rich sulfide-minerals after the GOE, coupled to climate-induced changes in riverine transportation patterns, would have influenced marine As concentrations during the course of the chemical evolution of the Early Proterozoic oceans. Further, >70% of As in the oxic surface ocean would have existed mainly in the As(V) oxidation state—the most mobile As species in oxidized waters^12^,^13^,^14^.

As(V) impairs phosphate (PO_4_^3−^) uptake and disrupts the synthesis and function of energy-conserving adenosine triphosphate, gene-coding nucleic acid molecules and cell membrane phospholipids^15^,^16^,^17^,^18^,^19^. The spread of protective mechanisms against As(V) damage throughout the tree of life^14^,^15^,^16^,^17^,^18^,^19^ therefore implies an early contact with harmful concentrations. This includes protection in the *Cyanobacteria*^18^,^19^,^20^, the architects behind Earth’s oxygenation^1^,^10^,^11^. Specific channeling of PO_4_^3−^ into cells—an adaptation for combating high environmental As(V)/PO_4_^3−^ molar ratios^19^,^20^,^21^,^22^—is widespread in the *Cyanobacteria*^19^,^20^,^21^. Coupled to the limited occurrence of less specific PO_4_^3−^ channeling characteristic of low As(V)/PO_4_^3−^ habitats in most *Cyanobacteria*^19^,^20^, these observations point to an evolutionary response to persistent As(V) threat in the deep past. As manifested by *Cyanobacteria* growth in the modern oxidized ocean surface^19^, microbial activity in high As(V)/PO_4_^3−^ conditions is tied to persistent expression of detoxification responses and elevation of specific PO_4_^3−^ scavenging^12^,^17^,^18^,^19^,^20^,^21^,^22^. Consequently, As(V) stressed *Cyanobacteria* down-regulate photosynthesis^12^,^14^,^19^ and therefore organic carbon and oxygen production.

The redox behavior of As (refs 12, 13, 14) suggests the distribution of As(V) would have been limited by several orders of magnitude in the oxygen-poor Archean Oceans^1^,^2^,^3^,^23^, with most of the As existing as As(III). As a consequence, exposure of extant life to the same level of As(V) toxicity seen in modern oxygen-rich aquatic environments would have been limited^12^,^13^,^14^. Therefore the GOE ushered in broad scale sensitization of microbial life to the effects of As(V) toxicity when surficial oceans first became permanently oxygenated as today. This view is consistent with proposals on adaptive physiological innovation in marine life, triggered by upheavals in levels of dissolved redox-sensitive elements after the GOE^7^,^24^. It also resonates with the opinion that changes in ocean chemistry constrained evolution^8^. However, how toxic redox-sensitive trace metal(loid) levels relate to ocean productivity, oxygen accumulation, climate and the timing and radiation of animal life remains obscure.

Fe(II) oxidation to Fe(III) was common in the Fe(II)-rich Precambrian oceans^23^, while Fe sulfides became more widespread in middle Proterozoic waters restricted to the continental margins^1^,^4^,^9^,^11^. Fe(III)(oxyhydr)oxides and Fe sulfide-rich solutions formed in marine and freshwater systems strongly bind and precipitate As from the water column^12^,^13^,^25^,^26^,^27^,^28^,^29^,^30^,^31^,^32^. This relationship is exemplified by a ~130,000 year (Ka) linear relationship between sedimentary As and Fe in an Fe(III)(oxyhydr)oxides-rich hydrothermal deposit in the East Pacific Rise (Fig. 1a). Although post-depositional diagenesis leads to some loss in sedimented As, the temporal and spatial As/Fe molar ratios are generally proportional to the original signals, since the proportion of lost As is relatively constant^26^. Further, Si, a prominent constituent in the early oceans^6^ does not appear to interfere with As coprecipitation. For example, marine hydrothermal As-rich Fe(III)(oxyhydr)oxides are reported to selectively exclude Si (ref. 27)while Si is not a strong component of sulfide-rich minerals. Thus the As/Fe molar ratio is a sensitive indicator for tracking spatial and temporal marine As variability (Fig. 1b). However, if the goal is to measure absolute dissolved As concentrations in past oceans, which was not the purpose of this study, corrections must be made for diagenetic As loss. Averagely, ~25% of the original As/Fe content in freshly precipitated Fe-rich marine sediments is lost to diagenesis^26^. Here we use the As/Fe molar ratios in sedimentary marine deposits to demonstrate that the geological As cycle is likely influenced by climate variability and coincidental with Precambrian oxygenation history.

To unravel the relationship between extreme climate and sedimentary As concentrations, we examined the link between As and continental Mn weathering in a submarine core from the crest of the Lomonosov Ridge, central Arctic Ocean. The core documents ~700 Ka of recent Earth climate history^33^. The brown colored interglacial sediment sequences of relatively high Mn content, alternate with glacial deposits of low Mn content, synchronized to changing weathering patterns, ocean ventilation and riverine delivery of sediments from the surrounding Siberian hinterlands^33^,^34^,^35^. Cyclical blocking and unblocking of continental weathering by the expansion and contraction of polar ice sheets during glacial and interglacial periods, respectively, are assumed to control the Mn oscillations^33^,^34^,^35^. Thus non-hydrothermal sediments delivered during the melting of ice and chemical weathering has been linked to a burst of Fe, Mn, Co, Mo, Ni, P and As in interglacial sediments from a 130 Ka record^34^. Diagenetic transformation and hydrothermal influence on the sediments are minor^35^, presenting an excellent opportunity to test if Mn weathering covaries with As in a climate sensitive area. This relationship is used to demonstrate a coupling between the marine sedimentary Mn and As reservoirs during glacial-interglacial cycles and to predict a connection between the two variables, linked to continental weathering. We confirm covariability between As and Mn in the Lomonosov Ridge^33^ through sequential acid extractions (Fig. 2a–h) as previously performed for the Mendeleev Ridge^34^. The Mn-As couple is then used as a proxy to interpret the contribution of climate-controlled continental weathering to Proterozoic sedimentary marine As concentrations, in combination with calculated chemical indices of alteration (CIA).

## Results and Discussion

### Controls on marine sedimentary As concentrations

Major controls on ocean chemistry are seafloor hydrothermal input and continental riverine runoff, coupled to continental building driven by tectonic activity throughout the Proterozoic^1^,^2^,^3^. With the motivation that the GOE changed Earth’s weathering patterns^4^,^6^,^9^,^10^,^11^, we investigated whether variations in weathering intensities would in addition to tectonic activity, influence sedimentary As levels. This hypothesis is tested using the chemical index of alternation: 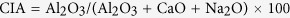
—a widely applied index that reveals subtle changes in past weathering fluxes^36^,^37^. Increasing CIA values imply intensification of chemical weathering of rocks and selective liberation of easily dissolvable CaO, Na_2_O and metals/metalloids into solution^36^,^37^. The broken rock particles, enriched in the poorly soluble Al_2_O_3_ fraction, sink to the seafloor as weathered sediments. Physical rock weathering, for example by moving ice and wind not accompanied by chemical weathering, leaves no net chemical change in the composition of sediments compared to parent rocks and a low CIA index.

Reference CIA indices for ancient soils representing upper continental crust weathering processes^36^ and CaO content, confirm changing weathering trends in our data (Fig. 3a–c). The CIA index for the ~3.8 Ga BIFs is consistent with a deep-sea depositional origin^38^ and an immature/anoxic early Archean continental crust characterized by reduced chemical weathering. The weathering indices suggest a switch to low and high chemical weathering during Proterozoic icehouse and greenhouse periods, respectively (Fig. 3a–c). For example, elevated icehouse CaO values agree with proposed coverage of continents by ice, reduced chemical weathering and riverine transportation of sediments to the oceans^39^,^40^,^41^,^42^,^43^,^44^,^45^. The result is abrasion of country rocks by moving ice but without significant chemical decomposition of resultant particles^37^. The discharge of this eroded debris into oxidizing environments during greenhouse periods would increase chemical weathering^37^. Sedimentary CaO deficits and the deposition of cap carbonates during greenhouse climates^39^,^40^,^41^,^42^,^43^,^44^,^45^ support this view. Further, CIA indices reported for glacial (<70) and postglacial sediments (>70) below and above these cap carbonates (refs 43,45 and references therein), respectively, are comparable to those obtained in this study (Fig. 3a).

### Marine sedimentary As dynamics and Fe mineralization

**Figure 1:**
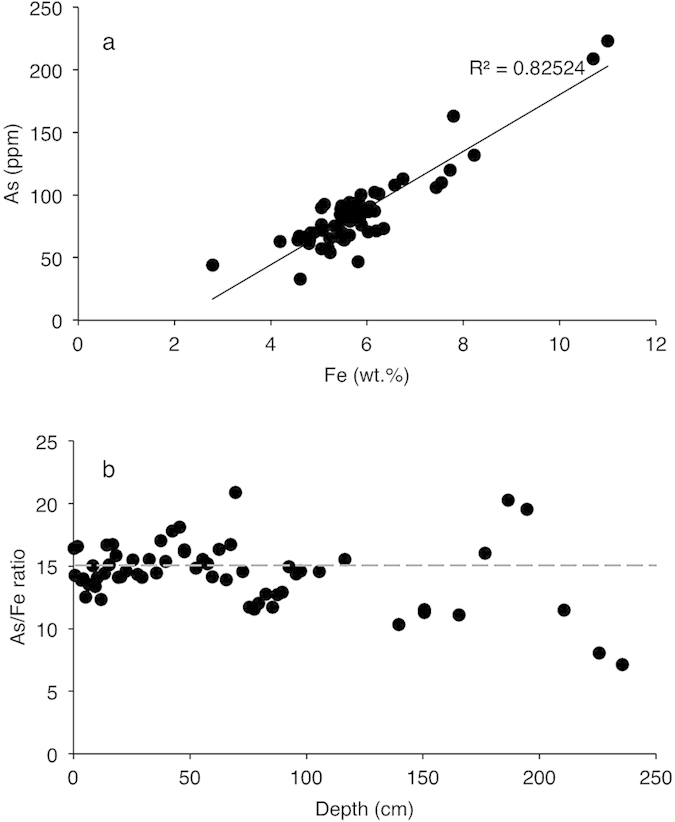
Sedimentary As and Fe dynamics through a 130,000 year long interval for a core from the East Pacific Rise hydrothermal vent field^26^. (**a**) Correlation between As and Fe. (**b**) Downcore As/Fe variability.

The sedimentary lithologies analyzed in this study are mainly from banded iron formations (BIFs) and organic carbon-rich black shales. Since Fe(III)(oxyhydr)oxides (abundant in BIFs) and Fe sulfides (abundant in black shales) are key marine sedimentary As sinks^12^,^13^,^26^,^27^,^28^,^29^,^30^,^31^,^32^, we use the As/Fe molar ratio to correct for potential differences arising from the precipitation of arsenic as As-sulfides, Fe sulfides, Fe-Mn oxyhydroxides and potentially with organic matter. This normalizes the data to marine Fe fluctuations through Earth history. This ratio is permissible because of the strong linear relationship existing between marine sedimentary Fe content and As (refs 12, 13, 14,25,26,29,31, Fig. 1).

Hydrothermal As-sulfides (e.g., realgar and orpiment) do not appear to strongly modify seafloor sedimentary As/Fe molar ratio, because pure As-sulfides precipitate near the anoxic vents, while Fe(III)(oxyhydr)oxides in spreading plumes are responsible for widespread dispersal of hydrothermal As in seafloor sediments^26^,^29^. However rapid precipitation of Fe sulfide minerals may result in a lag behind As precipitation by Fe(III)(oxyhydr)oxides if sulfate limitation induces low microbial sulfate reduction and thereby low sulfide production^28^. Such a case would be expected in the mainly sulfate-poor ferruginous Archean oceans^23^. However, buried As/Fe molar ratios would not have been impacted, because coprecipitation with the voluminous Fe(oxyhydr)oxides present in the ocean would quantitatively remove As, proportionally equal to sea water concentrations^12^,^13^,^14^,^25^,^26^,^29^. The binding of As(III) and As(V) on Fe(III)(oxyhydr)oxide and pyrite show similar high uptake patterns, with little discrimination as to the redox state of arsenic^12^,^13^,^25^,^26^,^27^,^28^,^29^,^30^,^31^,^32^. These processes tend to occur in marine hydrothermal settings where for example hydrothermal As(III) and Fe(II) is oxidized to As(V) and Fe(III)(oxyhydr)oxides, respectively, in the presence of oxygen and coprecipitated^25^,^26^,^27^,^29^,^31^. Abiotic conversion of As(III) to As(V) is extremely slow, but is accelerated by the oxidation of Fe(II) to Fe(III)(oxyhydr)oxides^32^. Thus oxygenation of the Fe(II)-rich Early Proterozoic ocean surface would have amplified the concentration of As(V) as a consequence of Fe(II) oxidation. In the Proterozoic, sulfidic conditions likely intensified near the ocean margins^1^,^4^,^9^,^10^,^11^, perhaps occupying ~1–10% of the global Mesoproterozoic seafloor^4^, while ferruginous conditions persisted on 30–40% of the open ocean floor^4^. These sedimentary sulfide levels are interpreted to reflect redox weathering of continental sulfide rich minerals and reprecipitation in mid-sulfidic waters^1^,^4^,^9^,^10^,^11^.

Since the chemistry of As closely follows that of sulfur^12^,^13^, oxidative dissolution of continental As-sulfides and reprecipitation on the seafloor should intensify over time, given that seawater sulfate concentrations increased from the Archean to present times^1^,^4^,^9^,^10^,^11^. We should therefore observe a steady increase in As concentrations in black shales from the Archean and onwards. However, as we show below, this is not straightforward. Our data instead suggest a complicated As cycle tuned to climate (Figs 4 and 5), potentially related to the blocking and unblocking of continental weathering and changes in the mechanism of supply of subaerial volcanic As to the oceans during glacial and interglacial periods (Fig. 6).

### The marine sedimentary arsenic record

Six distinct sedimentary As enrichment stages (Fig. 4, supplementary Table 1) are recognized on the basis of at least one order of magnitude change in the As/Fe molar ratio relative to reference ~^3^.^8^ Ga BIFs from West Greenland^38^. The stages are statistically supported by analysis of variance—ANOVA (*p* < 0.05) (Fig. 5a,b, supplementary Table 2). Stage I-II averages an increase of about one magnitude from ~3.8–2.5 Ga. A drop appears between ~2.49–2.1 Ga, when As/Fe molar ratios fall below ~3.8 Ga values (stage III) and coincides with a series of severe global glaciations (Huronian icehouse) believed to have punctuated this ~300 million year interval^39^,^40^. This then transitions into sedimentary rocks richer in As than at any time before this period (stage IV). A decline thereafter and through the middle Proterozoic ends in a second minimum during the Neoproterozoic global glaciations (Cryogenian icehouse) that lasted from ~0.85–0.58 Ga (refs 41, 42, 43, 44, 45) (stage V). A periodical but low amplitudinal oscillation as the signal declines into the Cryogenian icehouse is statistically supported by ANOVA (Fig. 3b, supplementary Table 3). Stage IV overlaps the Cryogenian ice age, the end marked by a return to high sedimentary As concentrations (stage VI), coming up to the Cambrian explosion at ~0.542 Ga, when most animal lineages appeared in the fossil record^46^.

Apparently, CIA indices in postglacial Paleoproterozoic sediments tend to intensify upwardly over a 1 km stratigraphic profile, away from glacial deposits^45^. Some have therefore suggested a slow emergence from the Huronian glaciations^43^,^45^ after ~2.2 Ga when they are officially believed to have ended^39^,^40^, although potential remnants of glacial sediments extend the minimum age to >2.09 Ga (ref. 40). This would explain the sudden drop in As concentration during glacial periods, especially at the onset of the GOE, linked to the blockage of continental As supply. Already by 2.7 Ga, we register significant increases in marine As concentrations (Fig. 4), consistent with increasing chemical weathering at this time, compared to earlier periods^1^,^10^. The sudden puzzling decline in As concentrations during the Huronian glaciation interval (2.4–2.1 Ga) is rather dramatic. The lowest As/Fe molar ratios recorded at this time are several magnitudes lower than 2.9–2.5 Ga and 2.1 Ga minima (Fig. 4). A similar situation is observed before and after the Cryogenian glaciations. We propose that these repetitive patterns reflect fluxes tuned to Proterozoic icehouse-greenhouse climate variability. It may be argued that the icehouse As/Fe dynamics are due to changes in marine Fe content without a corresponding change in As concentrations. However, hydrothermal As, which is positively correlated to sedimentary Fe dynamics^12^,^13^,^25^,^26^,^27^,^29^, would have been the predominant source of As in Proterozoic submarine waters overlain by thick ice sheets during the Huronian and Cryogenian glaciations, as would have been in the Archean.

**Figure 2:**
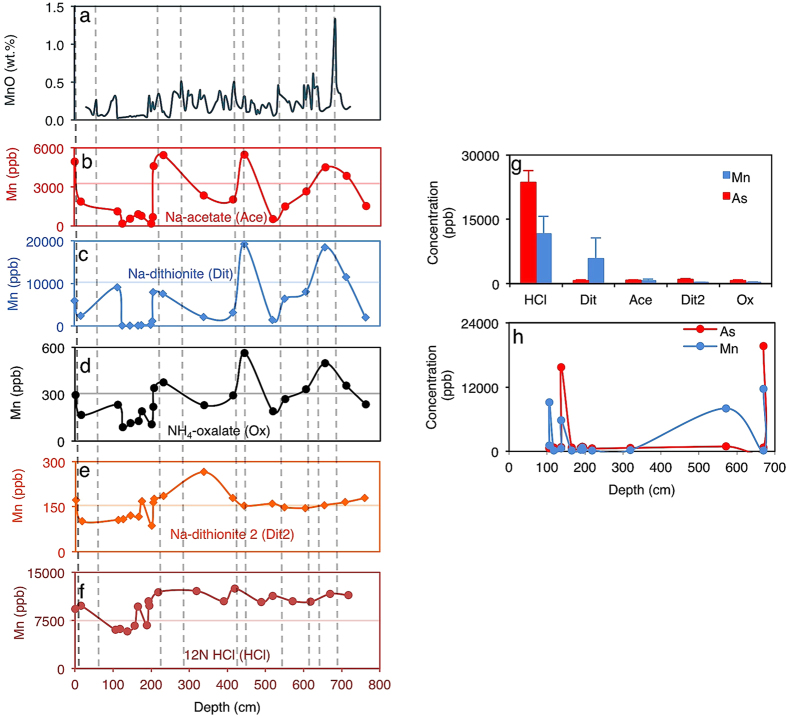
Arctic Ocean sediment Mn and As concentrations for the Lomonosov Ridge (1003 m water depth, 87°05.9′N, 144°46.4′E). (**a**) High resolution total MnO data showing low glacial and high interglacial oscillations, adapted from ref. 33. (**b–f**) Subsampled Mn fluctuations in sequentially extracted Fe-Mn fractions. Stippled gray lines are potential warm intervals (interglacials) as demonstrated in ref. 33. (**g**) Average Mn and As concentration in sequential extracts. (**h**) Mn and As covariation down the core. The decoupling between As and Mn seen at ~100 cm is associated to diagenetic Mn overprinting^35^ and is therefore considered anomalous. Because the ICP-OES detection limit for Mn was 22 times above that of As, only points where both Mn and As were unambiguously detected are included in the data.

**Figure 3:**
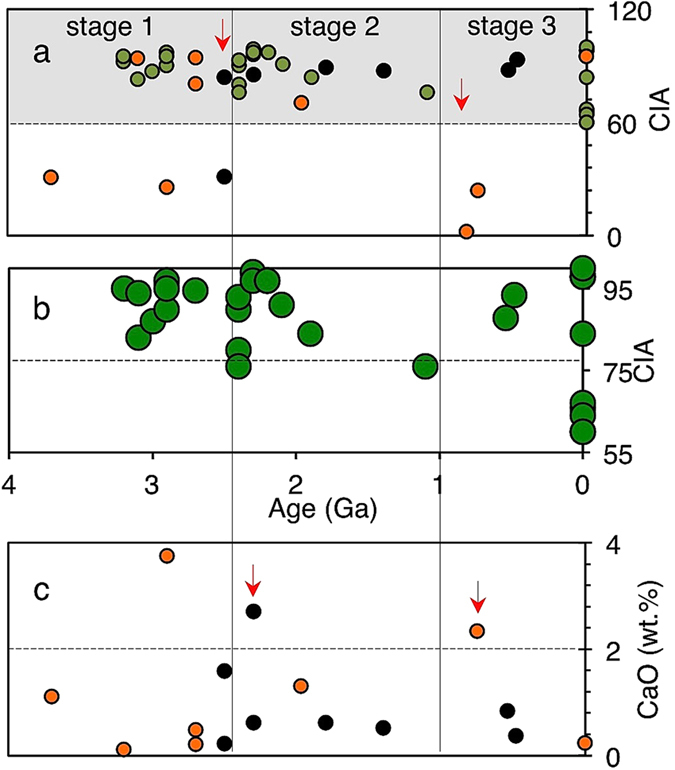
Chemical Index of Alteration (CIA). (**a**) CIA weathering profiles for shale deposits (black rings) and IFs (orange rings). Reference paleosol indices (green rings^36^. (**b**) Scaled up paleosol data. (**c)** Fluctuating CaO concentrations in shales and IFs in panel a. CIA and CaO data are averages for specific formations (comprising a total of more than 800 data points), calculated from values provided in ref. 36 and from major elemental concentrations in references given in supplementary Table 1. Red arrows correspond to icehouse periods. The recorded high CIA indices for recent hydrothermal sediments at Milos correspond to pervasive shallow submarine hydrothermal leaching of seafloor basement rocks, common for the Hellenic volcanic arc, Greece^25^.

**Figure 4:**
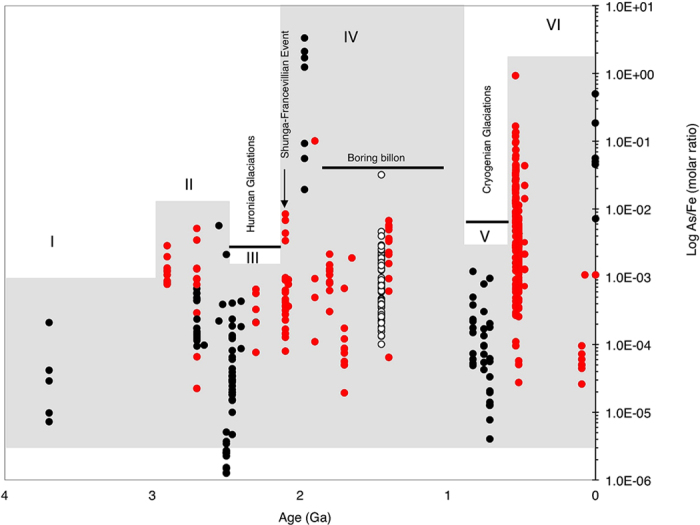
Sedimentary arsenic dynamics through geological time, showing trends in shales and IF deposits (see supplementary Table 1 for source of data). Red filled rings, IFs. Black rings, shale estimates. Unfilled rings, a mid-Proterozoic siliciclastic/sandstone reference deposit. I–VI represents key stages discussed in the text. High marine As concentrations (log As/Fe ratios >0.001) are not typical of modern marine concentrations. They are for shallow submarine hydrothermal sediments at Milos where vent fluids can contain >3000 times more As than seawater^25^. We did not find any significant differences between As/Fe molar ratios for shales and IFs of coeval ages, and between sulfidic and non-sulfidic samples.

**Figure 5:**
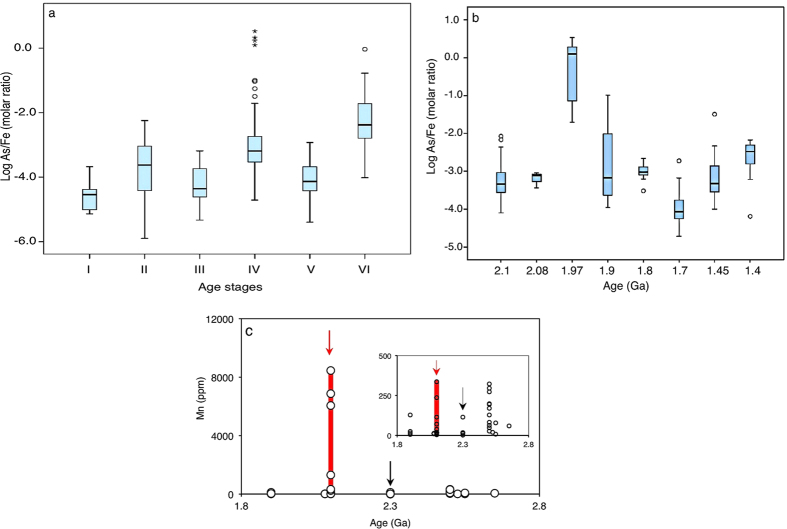
Box and whisker plots of log transformed As/Fe molar ratios. (**a**) Box and whisker plots grouped according to stages I–VI. One Way Analysis of Variance (ANOVA) confirms statistically significant differences (*p* < 0.05) between the stages (supplementary Table 2). (**b**) Expanded stage IV box and whiskers plots progressing from about ~2.1 to 1.4 Ga. ANOVA suggests significant differences between the groups (*p* < 0.05). (**c**) Mn concentration in sediments reported before, during and after the Paleoproterozoic Huronian glaciations^39^ (see supplementary Table 1). Red and black arrows indicate Huronian and post-Huronian glaciation sediments, respectively. Inset shows variations with extreme icehouse values omitted.

A slow emergence out of the Huronian glaciation would also explain the later stepwise increases in As concentrations seen after ~2.2 Ga (Fig. 4). This is consistent with reported enhanced continental weathering following the withdrawal of the extensive ice sheets covering land^43^,^45^. A majority of the analyzed sediments extending from the late Archean onwards, are shallow continental margin deposits, implying augmentation of the hydrothermal As source with supply from land. For example, elevated As levels are encountered in black shales composed predominantly of land-derived materials in the ~2.086 ± 0.006 Ga FD formation of the Francevillian basin analyzed here. A part of a 35,000-km^2^ sedimentary package, the FD formation is apparently characterized by no hydrothermal or metamorphic influence^47^,^48^,^49^. The average value for modern deep-sea authigenic As/Fe ratios for ferromanganese deposits is estimated to be ~11.10 × 10^−4^ and is anticipated to increase with hydrothermal activity and possibly when As is augmented from other sources^29^. In Franceville, As/Fe molar ratios exceed this authigenic value. Because of the absence of hydrothermal influence in these sediments, additional As must have originated from changes in processes occurring on land. Similarly, post-Cryogenian values respect this trend, exceeding expected seawater authigenic As/Fe molar ratios. The values for the icehouse sediments are either several magnitudes below expected seawater authigenic As/Fe molar ratios or are slightly higher, suggesting dilution of authigenic As/Fe molar ratios in basins cut off from continental supply and instances where hydrothermal As influx was prominent, respectively. The link between weathering and marine sedimentary As content during glacial-interglacial intervals, is further inferred from our analysis of Mn and As variability in the Arctic sediments deposited during well-defined glacial-interglacial cycles (Fig. 2). As expected, we observed a coincidental increase in both Mn and As in the post-Huronian sediments (interglacial, Fig. 5c) strongly influenced by chemical weathering (Fig. 3).

### Sedimentary As dynamics and redox-sensitive Paleo-proxies

Similar to Cr sedimentary data through geological time, sedimentary As concentrations are generally low during the Archean^9^. We record low sedimentary As concentrations in the Dales Gorge. However, the decline in As concentrations during the Huronian glaciations deviates markedly from that of Cr reported for the same Timeball Hill sediments we have analyzed at ~2.3 Ga. Further, there is not a corresponding increase in Cr seen after the Huronian glaciations as with As. Nonetheless, a similar decreasing sedimentary trend is tracked after ~2.1 Ga. Although both As and Cr are redox-sensitive, their environmental mobility is controlled differently by factors such as pH and degree of binding Fe(III)(oxyhydr)oxides and sulfidic minerals. For example, As is more readily co-precipitated with sulfides^12^,^13^ than Cr which is more readily precipitated under anoxic-ferruginous conditions as (Fe,Cr)(OH)_3_ minerals^4^,^9^. Similarly, Mo is mostly precipitated in response to sulfidic conditions^1^,^4^,^47^. On the other hand, As species strongly form solid phase minerals with both Fe(III)(oxyhydr)oxides and sulfidic minerals to about the same magnitude^12^,^14^,^30^. For instance, we did not find any significant differences in coeval sedimentary As levels associated with non-sulfidic IFs and sulfidic shales. Therefore, different redox-sensitive trace elements are not expected to show coeval sedimentary trends at all times. Rather their sedimentary distribution likely reflects their unique redox chemistries as well as pH behavior, affinities to different scavengers (organo-metallic) and diagenetic behavior. In the case of As, both the environmentally important As(III) and As(V) oxidation states coprecipitate with Fe(III)(oxyhydr)oxides and sulfide-rich minerals, in acidic and basic conditions^12^,^13^. Cr precipitates mostly as Cr(III) with Fe(III)(oxyhydr)oxides and to some extent with sulfides. Cr(VI) is mobile under neutral to alkaline conditions^9^. Therefore the sedimentary As trends seen are not likely due to redox or pH changes, but probably to an intrinsic factor affecting the supply of As to the oceans. Low As signals are consistently seen both during the Huronian and Cryogenian glaciations, which seems to provide support for the fact that glaciations somehow diminish the amount of As present in the oceans.

### Arsenic enrichment in glacial sediments

**Figure 6:**
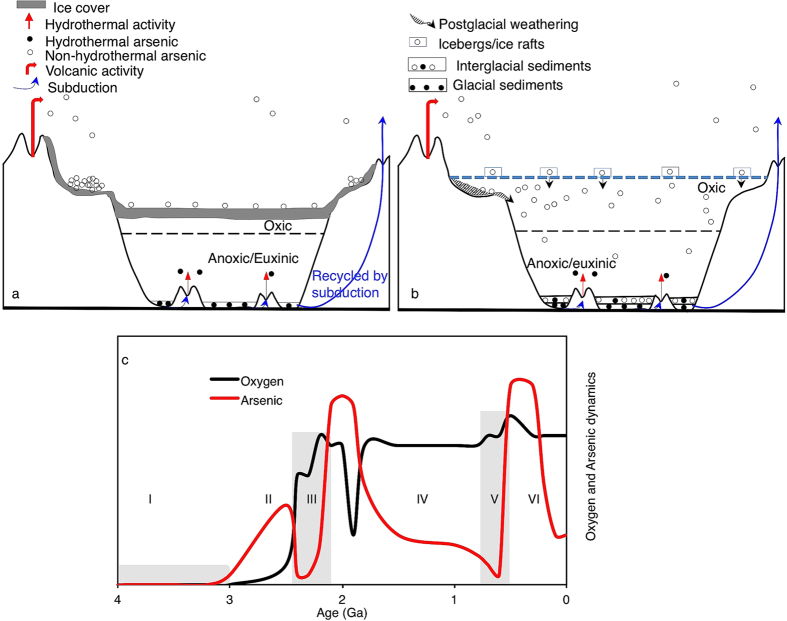
Conceptual model of Proterozoic icehouse-greenhouse marine As cycling. (**a**) Snowball Earth scenario characterized by global ice sheets and low sea levels. (**b**) Greenhouse Earth characterized by ice retreat, sea level rise and massive As flux into the ocean. (**c**) Model of temporal sedimentary As inventory from Fig. 4, superimposed on oxygen dynamics through Earth History^1^. Stages I–VI are defined according to Fig. 4.

Available evidence suggests the global ice sheets that persisted for millions of years captured and concentrated atmospheric pollutants falling out of the subaerial volcanoes (Fig. 6a) that became prominent at the GOE (refs 2,3). For instance, >40 fold increase in As concentrations are recorded in Arctic and Antarctic ice cores deposited during the recent coldest four ice ages^50^,^51^. A volcanic source is suggested for As deposited in these ice cores^50^, together with the enrichment of a vast range of elements including Bi, Mn, Co and U. Moreover, Pleistocene glacial deposits are generally rich in As and constitute some of the major sources of modern As contamination problems in groundwater in Asia and North America (e.g., refs 12, 13, 14,52,53). Presently, volcanic gases emit ~17,150 tons of As per year to the atmosphere^13^. During the ~300, 60 and 15 million year duration of the Huronian and Cryogenian Sturtian and Marinoan ice ages, an incredible 5.1 × 10^12^, 1.0 × 10^12^ and 2.6 × 10^11^ tons of volcanic As, respectively, could thus have been deposited on the expansive global ice sheets. This proposition follows the hypothesis that long-term buildup of volcanic CO_2_ in the atmosphere terminated the Proterozoic icehouses^39^,^40^,^41^,^42^,^43^,^44^,^45^. Thus a burst of deglaciating metal/As-rich waters and intensified chemical weathering would have changed the trace elemental content of the oceans (Fig. 6b). These observations perhaps provide one further explanation for the differences recorded between Cr and As at different times. The principal source of Cr to the ocean is from the dissolution of chromite from ultramafic rocks^9^. The accumulation of volcanic As on surface ice would have cut off a strong direct source of As to the oceans. However, the supply of physically eroded sediments to the oceans through under-ice liquid channels and by icebergs and ice rafts could still account for the Cr record even if this weathering was not accompanied by strong chemical weathering, as our data suggest.

### Arsenic, ocean productivity and atmospheric oxygen dynamics

In contemporary surface oceans, As(V) interference with *Cyanobacteria* productivity induces rapid mitigating responses^12^,^19^,^20^,^21^. Our data cannot enable explicit unraveling of similar processes in the Precambrian oceans characterized by varying temporal As dynamics. This is hampered by the lack of suitable proxies to directly quantify biological As detoxification and metabolic pathways through time. How As contributed in defining trends in Precambrian primary productivity and thereby oxygen dynamics, is therefore a matter opened for discussion. This is especially crucial as it is established that marine As(V) maxima coincide with photosynthesis minima and vice versa^12^,^14^,^19^. However, based on the conservative redox behavior of As in modern oceans, we speculate that life likely encountered widespread As(V) exposure for the very first time when surface oceans became permanently oxidized during the GOE.

We uncover an inverse association between sedimentary As content and interpreted free oxygen levels^1^ in the Early Paleoproterozoic (Fig. 6c). Elevated Paleoproterozoic As dynamics coincide with a suggested sharp plunge in atmospheric oxygen content in the aftermath of the Huronian icehouses, and a rebound also consistent with weakening sedimentary As intensity. All of these are within the bounds of a short-lived acute crash in primary productivity seen as a severe oxygen paucity during the so-called Shunga-Francevillian negative δ^13^C isotope excursion, after ~2.086 ± 0.006 Ga (refs 1,47,54). A modern marine As resident time of ~100,000 years, sedimentation rates of ~46,400 tons year^-1^ and long-term subduction and recycling of ~38,200 tons year^-1^ into the biosphere by tectonic processes^13^,^55^, also fit inside this oxygen scarcity window. These processes would have eventually diluted the As-rich sediments, as evident in the subsequent steady decline in sedimentary As content (Fig. 6c, IV), paralleled by an ocean productivity stasis in the mid-Proterozoic known as the boring billion^1^,^4^. A recent study infers that atmospheric oxygen content may have stayed at ~0.1% of present day levels during this duration^56^. Thus, biogeochemical evidence suggests that habitable oceanic conditions may have deteriorated during this boring billion period, reflected by stasis in primary productivity and thus overall low oxygen concentrations^1^,^4^. We propose that one contribution to this was the reorganization of the marine elemental cycles following the massive release of glacial sediments and elements captured in the glacial ice sheets into the oceans. This new wave of redox-sensitive elements may have severely perturbed primary productivity until when life had developed the necessary protective mechanisms.

Microbial As(III) oxidation and As(V) reduction for energy gain is considered an ancient process^16^,^17^. However no modern obligate As(V) reducers are known, with lineages often affiliated to both Fe(III) and sulfate-reducing microorganisms^16^. In the mainly anoxic Archean oceans, Fe-respiring bacteria capable of reducing both Fe(III) and As(V) to Fe(II) and As(III), respectively, would have been prominent, while the activity of sulfate reducers would have been limited because of low sulfate content. Therefore the extremely high Fe content of the Archean oceans^23^, coupled to low redox transformation of dissolved As(III) to As(V), may have limited microbial As(V) reduction. Following the transition to oxidized Proterozoic sulfate-rich oceans after the GOE, As(V) precipitation likely increased, but the growing sulfate content in the anoxic bottom waters may have presented a competitive challenge on microbial As(V) reduction. Chemoautotrophy and photoauthotrophic oxidation of As(III) may also have generated organic matter in the ancient oceans^16^. However, the magnitude of how these various processes globally affected the ancient marine As cycle is unknown, and cannot be quantified from our data. Our historical As trends may provide a timeline for potentially constraining when such processes likely became prominent in deep time.

### Arsenic and the Shunga-Francevillian negative δ^13^C_org_ excursion

Negative δ^13^C_org_ excursions after ~2.1 Ga in Franceville (Gabon, Central Africa) and Shunga (Karelia, Russia) (refs 1,47,54) contrast pronounced worldwide positive δ^13^C records linked to up to 22 times oxygen liberation, relative to modern atmospheric concentration, during the so-called Lomagundi Event that climaxed at ~2.2 Ga (refs 1,11). This Shunga-Francevillian event is instead allied to oxygen scarcity^1^,^47^,^54^, and has been attributed to global-scale oxidation of rifted and uplifted ancient organic matter^1^,^11^,^47^,^54^. The termination of the Cryogenian icehouses by comparable negative δ^13^C shifts^41^,^42^,^43^,^44^,^45^ linked to irreversible increases in atmospheric oxygen content^1^ thus presents a perplexing paradox. Rifting, uplifting and weathering of buried organic matter consume oxygen. It would intensify an icehouse effect if CO_2_ consumption by exposed silicates offsets production by organic matter remineralization. This would be inconsistent with hypothesized CO_2_ buildup providing an escape mechanism into succeeding greenhouse climates^39^,^40^,^41^,^42^,^43^,^44^,^45^. Based on our results, we provide the following speculative alternatives: (I) Mass elimination of biomass following global scale postglacial perturbation of marine biogeochemical elemental cycles. (II) Ocean-wide oxidation of exterminated biomass without corresponding oxygen liberation by stressed primary producers after ~2.1 Ga. Such a mechanism could explain both observed oxygen deficits and the Shunga-Francevillian negative δ^13^C isotope excursion. The isotopic excursions at this time have been previously linked to a potential mass extinction event attributed to a possible impact event (see 43 and references therein). Indeed, Phanerozoic mass extinctions followed by negative δ^13^C isotope excursions, linked to catastrophic disturbance of the carbon cycle, support this alternative hypothesis^57^. (III) Primary producers overcame postglacial physiological distress experienced for the first time after ~2.1 Ga, enabling their success in similar conditions in the Ediacaran-Early Cambrian interval. Consequently, oxygen liberation exceeded consumption by organic matter oxidation.

A combination of chemical weathering proxies, including CIA, CaO concentrations and covariations in sedimentary As and Mn concentrations coupled to glacial-interglacial cycles, suggest climate induced variation in chemical weathering fluxes imposes a hitherto unknown strong oscillation on marine As concentrations. Consistent sedimentary As minima appear during posited Snowball Earth intervals. High amplitude anticorrelations between Early Proterozoic sedimentary As concentrations and atmospheric oxygen content are less evident in the As-rich post-Cryogenian-Early Cambrian oceans. In accordance with evolutionary principles of natural selection, the Precambrian-Cambrian boundary likely marks a period of widespread biological stabilization to the negative feedback consequences of calamitous global-scale icehouse-greenhouse reorganization of marine elemental cycles. Widespread mitigation practices against prohibitive As(V) stress would have stimulated cells to capitalize on the return of postglacial nutrient-rich conditions in the Ediacaran, enhancing both metabolism and innovative processes (e.g., ref. 6). As seen in the modern oxidized ocean surface and in As(V)-rich environments, evolutionary adaptations for combating sudden hazardous As(V) availability in the ancient oxygenated ocean surface would include the following two mechanisms. (I) The innovation of rapid intracellular detoxification mechanisms^12^,^14^,^15^,^16^,^17^,^18^,^19^, which continuously converts and extrudes intracellular As(V) as As(III)^12^,^13^,^14^,^16^,^17^. When released into the environment, As(III) is again oxidatively transformed to As(V). Thus As(V) detoxification is a temporal relief mechanism that has probably been ongoing since the time cells first became exposed to large amounts of As(V) in the Early Proterozoic. (II) The stabilization of high affinity specific phosphate scavenging, which is strongly induced by increasing dissolved As(V)/PO_4_^3–^ levels, would have ensured that marine organisms sustained their obligatory phosphate requirements^19^,^20^,^21^,^22^. This protective mechanism is spread across a wide range of modern *Cyanobacteria*^20^,^21^,^22^ and probably has not changed since the Precambrian-Cambrian boundary. The exact extent to which the As(V)/PO_4_^3–^ levels impacted life remains to be evaluated. For this, coeval dissolved concentrations of As(V) and PO_4_^3–^ are required, which is beyond the scope of the current study. However, the magnitude of As enrichment in postglacial sediments coupled to known redox conditions and the effect of As on extant life, suggest that postglacial enrichment of As in marine basins may have imposed a tremendous barrier to the activities and expansion of life, requiring strong adaptive responses. Additional adaptive responses to documented post-Cryogenian elevated levels of beneficial, yet toxic trace metals such as Cu and Zn (refs 7,8) associated with metalliferous oceans^58^, together produced physiologically robust communities that permitted increased oxygenation of the ocean-atmosphere system, from which complex life radiated. If the emergence of multicellurity is therefore linked to global glaciations, it is also possible that the purported ~2.1 Ga large colonial fossils of the Francevillian FB formation^59^ indeed represent first experimentation at multicellularity halted by postglacial decline in habitable environmental conditions.

## Methods

### Precambrian sediments

A suite of rock samples analyzed in this study was chosen to particularly bracket the Archean-Proterozoic transition and the Great Oxidation Event. Literature data were compiled for well-characterized marine geological formations with details of field investigations, including depositional setting, petrology, stratigraphy, trace and major element abundances and isotope geochemistry in several cases. A key aim of our study was to use representative BIFs and black shale samples that have been well characterized to test the central hypothesis of the paper, which is whether variations in climate fluctuation have influenced marine As concentrations linked to weathering patterns and changes in volcanic activity through geological time. For example we use the well-characterized Rapitan and Urucum BIFs deposited in the height of the Sturtian glaciations—the severest Neoproterozoic glaciations that occurred between ~0.75–0.7 Ga, together with a recently described Neoproterozoic BIF sample from the Shilu district, Western Hainan Province, South China. The latter was probably deposited at the onset of the Cryogenian glaciations, dated at ~0.83 Ga (supplementary Table 1 and references therein). The geological formations are generally suggested to have witnessed limited metamorphism (see references in supplementary Table 1). In cases where extensive metamorphism has been reported, previous interpretations have shown that the sediments still maintain the seawater chemical signature from which they formed (e.g., ref. 38). Black shales, aged ~2.6–2.2 Ga, were sampled from the Chuniesport Group, in the Transvaal Supergroup, South Africa. These include the Oaktree, Monte Cristo, Lyttelton, Eccles and Frisco formations, deposited in a shallow seaway and capped by a carbonate platform and overlain by the Penge BIFs. Black shale facies from the Timeball Hill Formation (~2.3 Ga), of the Lower Pretoria Group, are from a shallow to deep marine platform noted for displaying glacial influences. Black shales, aged ~2.1–2.084 ± 0.006 Ga, were obtained from the FB and FD formations of the Francevillian series, Gabon, Central Africa, of the same suite and stratigraphy as reported in ref. 47, 48, 49. Black shale units recently constrained to ~1.65 Ga from outcrops^59^ of the Vindhyan Super Group (central India, Jankikund) were also analyzed. The samples originate from a subtidal to supratidal marine environment. Pulverized and completely acid-digested solutions (using a mixture of HNO_3_, hydrofluoric acid-HF and oven temperatures >200 °C) were analyzed by Inductively Coupled Plasma-Optical Emission Spectroscopy (ICP-OES) for total elemental composition. Precision according to certified standards was estimated to be 3–5%, depending on element. Instrument sensitivity was 1 ppb. Samples and standards were measured three times and averaged. A Columbia River Basalt (BCR-2) certified reference material and blanks containing extraction reagents were digested according to the same procedure as for the geological samples and simultaneously analyzed. The samples include both sulfidic and ferruginous deposits, since As is commonly coprecipitated with Fe (oxyhydr)oxides and sulfides to about the same magnitude^12^,^13^,^14^.

### Chemical Index of Alteration (CIA)

CIA and CaO data were estimated from reported major elemental concentrations in references in supplementary Table 1. Samples were selected to minimize carbonates facies, which could bias the results towards lower values^39^,^40^,^41^,^42^,^43^,^44^,^45^.

### Weathering and marine sedimentary Mn and As concentration

A 722 cm core from the Crest of the Lomonosov Ridge, Central Arctic Ocean^33^ was sampled. The core covers at least four glacial-interglacial cycles recorded in the recent Pleistocene to the present Holocene interglacial. Mn signals in the core demarcate glacial-interglacial periods for the last ~700,000 years^33^,^35^. Fifty milligrams of sampled sediments were sequentially extracted with acids^47^,^60^ to obtain Fe-Mn fractions associated with 1) carbonates (sodium acetate), 2) Oxyhydroxides (sodium dithionite), 3) Fe as magnetite (ammonium oxalate), 4) Hematite-phases (sodium dithionite 2), 5) Sheet silicates (12M HCl). Solutions were measured for total elemental composition using ICP-OES as stated above. Because Fe sulfides are negligible or absent in Arctic Ocean sediments^33^,^34^,^35^ they were not analyzed. Furthermore, the sequential analysis provides a better means of resolving trends that may be unresolved because of masking by excessive detrital input^47^. This analysis aimed to establish how climate affects marine As concentrations and to draw analogies with intense Proterozoic climatic fluctuations.

### Statistical analysis

One-Way ANOVA was applied to the log-transformed As/Fe molar ratios using the IBM SPSS ver.22 software in order to identify statistically significant differences corresponding to different stages of As evolution in the sedimentary record.

## Additional Information

**How to cite this article**: Chi Fru, E. *et al.* Arsenic stress after the Proterozoic glaciations. *Sci. Rep.* **5**, 17789; doi: 10.1038/srep17789 (2015).

## Supplementary Material

Supplementary Information
