# Supplementary material for: Arsenic stress after the Proterozoic glaciations
Source: Sci Rep. 2015 Dec 4;5:17789. doi: 10.1038/srep17789 (PMC4669525; doi:10.1038/srep17789)
Supplement: Supplementary Information [file srep17789-s1.pdf]

Supplementary data
Arsenic stress after the Proterozoic glaciations

Ernest Chi Fru[1,2*], Emma Arvestål[2,3], [1]Nolwenn Callac, El Albani Abderrazzak[4], Stephanos Kilias[5], Ariadne Argyraki, Martin Jakobsson[1]

[1]Stockholm University, Department of Geological Sciences, SE-106 91, Stockholm, Sweden.
[2]Nordic Centre for Earth Evolution, Swedish Museum of Natural History, Department of Palaeobiology and Box 50007, SE-104 05, Stockholm, Sweden.
[3]Uppsala University, Department of Earth Sciences, Paleobiology, SE-752 36, Uppsala, Sweden.
[4]Institut de Chimie des Milieux et Matériaux de Poitiers (IC2MP)UMR 7285 CNRS - Université de Poitiers 5, rue Albert Turpin (Bât B35) 86022 Poitiers cedex.
[5]Department of Economic Geology and Geochemistry Faculty of Geology and Geoenvironment, University of Athens Panepistimiopolis Zographou 157 84 Athens, Greece.

Supplementary Table 1. Sources of data for the calculation of Fe and As molar concentrations.

Age (Ga)	Depositional environment	Rock type	Sample location	Lithology	Fe (molar)	As (molar)	As/Fe	References
3.8	Deep marine	Sedimentary	Isua, West Greenland	BIF	0.256965358	5.40565E-05	0.000210365	1
					0.61467644	BD	BD	
					0.279917339	BD	BD	
					0.207995285	8.67574E-06	4.17112E-05	
					0.23846202	1.73515E-06	7.27641E-06	
					0.138142651	4.00419E-06	2.89859E-05	
					0.123238775	1.20126E-06	9.74738E-06	
2.9	Averages of 802 Marine shale composites	Sedimentary	Superior province of the Canadian Shield	Black shale	0.130252364	0.001065714	0.001065714	2
					0.001127184	0.002883697	0.002883697	
					0.106456259	0.000774237	0.000774237	
					0.146533909	0.001979183	0.001979183	
					0.078902874	0.000828889	0.000828889	
					0.101446553	0.001198928	0.001198928	
					0.090174714	0.001237603	0.001237603	
					0.077650448	0.000869398	0.000869398	
					0.09518442	0.001349904	0.001349904	
2.9	Salobo iron oxides	Sedimentar	Carajas Mineral Province, Salobo, northern Brazil	BIFs, deposited in rift basin	0.182727179	0.000192201	0.001051846	3
					0.116722898	7.07406E-05	0.000606056	
					0.146746743	4.00419E-05	0.000272864	
2.7	Marine	Volcanic/sedimentary	Sandur superterrane. Western Dawhar, India	Black shales (associated with metabasalts, cherts, greywacke and BIF)	0.189503981	0.000173515	0.000915626	4
					0.024995142	7.34101E-06	0.000293697	
					0.095772201	0.000125464	0.00131003	
					0.01759862	3.06988E-06	0.000174438	
					0.187080983	0.000173515	0.000927485	
					0.185295616	4.13766E-06	2.233E-05	
					0.15060848	0.000200209	0.001329336	
					0.167569469	0.000112117	0.000669079	
					0.017981199	8.80921E-06	0.000489912	
					0.149970849	0.000774143	0.005161954	
					0.172415466	0.000600628	0.003483608	
					0.034687135	1.98875E-05	0.000573338	
					0.108779876	4.85841E-05	0.000446628	
					0.056621647	3.2167E-05	0.000568104	
					0.046547075	3.56373E-05	0.000765617	
					0.116350429	0.000109448	0.000940673	
2.699	Marine /hydrothermally-influenced	Metavolcanic/metasedimentary	Sukumaland Greenstone Belt, Geita,Tanzania	BIF (Magnetite-hematite-Chert)	0.113625873	1.60167E-05	0.00014096	5
					0.418923673	4.80502E-05	0.000114699	
					0.393035848	9.3431E-05	0.000237716	
					0.39163306	5.20544E-05	0.000132916	
					0.215136754	0.000100105	0.000465307	
					0.387042115	6.00628E-05	0.000155184	

					0.625898749	7.74143E-05	0.000123685	
					0.465470748	3.06988E-05	6.59521E-05	
					0.228782061	3.20335E-05	0.000140017	
					0.230950007	3.20335E-05	0.000138703	
					0.621690383	5.87281E-05	9.44651E-05	
					0.37199402	5.45904E-05	0.000146751	
2.55					0.022186409	2.18228E-05	0.000983611	This study
					0.00146638	2.13023E-05	0.014527133	
					2.08618E-05	0.003919778	0.005322189	
					0.110967857	6.28657E-05	0.000566522	
2.49	Marine	Sedimentary	Dales Gorge, Brockman Iron Formation, Western Australia	Hematite-rich BIF	3.426535231	5.20544E-06	1.51916E-06	6
					4.592852001	5.87281E-06	1.27868E-06	
					4.640290626	5.87281E-06	1.26561E-06	
					5.438116035	2.00209E-05	3.68159E-06	
					3.497117378	1.78854E-05	5.11432E-06	
					4.318982183	1.09448E-05	2.53411E-06	
					5.09744525	1.32138E-05	2.59224E-06	
					5.227685379	1.18791E-05	2.27234E-06	
					5.419970275	1.4682E-05	2.70887E-06	
					2.176563703	7.47448E-06	3.43407E-06	
					3.249578118	4.80502E-06	1.47866E-06	
					4.234248545	1.04109E-05	2.45873E-06	
					4.276615453	1.06778E-05	2.49679E-06	
2.4	Shallow passive Marine	Sedimentary	Cauĉ formation, Quadrilátero Ferrífero, Minas Gerais, Brazil	BIF (dolomite-rich and hematite-rich bands and quartz-rich bands and hematite-rich bands)	0.967249045	8.40879E-05	8.69351E-05	7
					0.707621016	0.000129469	0.000182963	
					0.14703488	6.4067E-05	0.000435726	
2.46	Relatively deep marine	Sedimentary	Kuruman Iron Formation, Transvaal basin in Griqualand West, South Africa	Microbanded siderite-rich BIF transitions to Griquatown granular iron formation (GIF)	0.50895113	1.76184E-05	3.46171E-05	8
					0.668063144<br>0.466063446	6.67364E-06<br>9.3431E-06	9.98954E-06<br>2.00468E-05	
					0.287386356	8.27532E-06	2.87951E-05	
					0.468913987	2.05548E-05	4.38349E-05	
					0.381454208	9.3431E-06	2.44934E-05	
					0.441056428	2.66946E-05	6.05242E-05	
					0.431338674	1.20126E-05	2.78495E-05	
					0.435873626	9.3431E-06	2.14353E-05	
					0.62582331	2.10887E-05	3.36975E-05	
					0.313429935	1.4682E-06	4.68431E-06	
					0.228043276	4.13766E-06	1.81442E-05	

					0.229209407	1.86862E-05	8.15246E-05	
					0.391949381	5.87281E-06	1.49836E-05	
					0.483166692	9.3431E-06	1.93372E-05	
					0.35800203	2.33577E-05	6.52447E-05	
					0.515300063	9.3431E-06	1.81314E-05	
					0.411903168<br>0.612995875	5.33891E-05<br>9.3431E-06	0.000129616<br>1.52417E-05	
					0.512319952	2.00209E-05	3.9079E-05	
					0.486405943	1.4682E-05	3.01847E-05	
					0.488349494	1.4682E-05	3.00646E-05	
					0.505193599	1.73515E-05	3.43462E-05	
					0.266136869	0.000108113	0.000406231	
					0.191763664	4.53808E-05	0.000236649	
					0.271967521	5.07197E-05	0.000186492	
					0.459973654	6.27322E-05	0.000136382	
					0.437817177	5.33891E-05	0.000121944	
2.3	Shallow seaway deposit	Sedimentary	Chuniesport Group, South Africa,(Oaktree, Monte Cristo, Lyttelton, Eccles and Frisco formations)	Black shales with glacial influences	0.052771063	2.58804E-05	0.000490428	This study
					0.052771063	2.58804E-05	0.000490428	
					0.488136807	8.59699E-05	0.000176118	
					0.052180141	2.53465E-05	0.00048575	
					0.037210135	4.85975E-05	0.001306029	
					0.033181126	2.52531E-05	0.000761068	
2.1	Relative deep marine continental shelf	Sedimentary with little or no hydrothermal influence	Francevillian Series, Republic of Gabon, Africa	Euxinic Black shales deposited at the end of the Lomagundi Event	0.042795416	0.000578338	0.013514015	This study
					0.0959484	0.00272685	0.028419968	
					0.004947085	8.05909E-05	0.016290586	
					0.005047279	8.3247E-05	0.016493445	
					0.02404659	3.07922E-05	0.001280522	
					0.033126683	0.000186995	0.00564486	
					0.040929301	1.87796E-05	0.000458831	
					0.050278665	9.38982E-06	0.000186755	
					0.043108523	0.000145886	0.003384153	
					0.081395203	9.33109E-05	0.001146393	
					0.00519757	0.000138945		
					0.050197257	0.000408293	0.00813378	
					0.016832613	8.01371E-05	0.004760824	
					0.037409982	0.000577404	0.015434479	
					0.008917277	0.000143216	0.01606055	

					0.044536289	0.00020408	0.004582331	
					0.036094934	0.000160968	0.004459581	
					0.033665226	0.000421641	0.012524519	
					0.027152608	0.000400419	0.014746965	
					0.028029307	0.000262675	0.009371426	
					0.044248231	0.000214758	0.004853478	
					0.032099693	0.000114827	0.00357719	
					0.055858225	0.000537362	0.0096201	
					0.120996932	0.000132619	0.00109605	
					0.032575615	0.000144685	0.004441499	
					0.036683574	0.000261874	0.007138719	
					0.031861732	8.54093E-05	0.002680623	
					0.019137078	7.27694E-05	0.003802535	
					0.055319682	5.8995E-05	0.001066438	
					0.019324942	2.19963E-05	0.001138235	
					0.038862797	3.81599E-05	0.000981913	
					0.055883274	6.54417E-05	0.001171043	
					0.154136139	0.000244656	0.001587271	
					0.044310852	5.35894E-05	0.001209396	
					0.412048344	4.34588E-05	0.00010547	
					0.003789059	2.71617E-05	7.17E-03	
					0.062780911	4.22041E-05	6.72E-04	
					0.055689856	4.90379E-05	8.81E-04	
					0.002369057	3.37953E-05	1.43E-02	
					0.045859074	3.57173E-05	7.79E-04	
					0.02886561	2.78024E-05	9.63E-04	
					0.048974841	9.13221E-05	1.86E-03	
					0.002463963	4.38725E-05	1.78E-02	
					0.046575342	9.11486E-05	1.96E-03	
					0.045465127	1.60835E-05	0.000353755	
					0.060291879	1.82724E-05	0.000303066	
					0.004492793	4.14433E-05	0.009224397	
					0.022956397	1.77252E-05	0.000772125	
					0.025463336	1.48956E-05	0.000584982	
					0.12275047	2.06616E-05	0.000168322	
					0.023189184	2.26236E-05	0.00097561	
					0.042492613	4.16035E-05	0.000979076	

					0.007882532	1.59767E-05	0.002026849	
					0.06072164	2.93774E-05	0.000483804	
					0.013322589	1.76852E-05	0.00132746	
2.083	Relative deep marine continental shelf	Sedimentary with little or no hydrothermal influence	Francevillian Series, Republic of Gabon, Africa	Euxinic Black shales deposited at the end of the Lomagundi Event		0.000374391		This study
					0.094996556	0.001247838	0.01313561	
					0.11206713	0.001768515	0.015780857	
					0.102235581	0.001918005	0.01876064	
					0.076886468	0.000879853	0.011443536	
					0.103325193	0.000446066	0.004317111	
					0.114809944	0.00238249	0.020751604	
					0.08135763	0.000736503	0.009052663	
					0.080330641	0.001200455	0.014943923	
					0.088471413	0.000975954	0.011031287	
					0.106042958	0.000189398	0.00178605	
					0.071826664	8.29534E-05	0.001154911	
					0.051732474	9.8236E-05	0.001898923	
					0.026125884<br>0.01041275	2.00076E-05<br>1.68309E-05	0.000765815<br>0.001616374	
1.97	Marine	Metavolcanic/ metasedimentary	Homestake Iron Formation. Lawrence County, South Dakota	IF	0.3018982	0.005878816	0.019472843	9
					0.366683223	0.62732243	1.710802105	
					0.344656315	0.03203349	0.092943285	
					0.380935928	0.80751078	2.11980735	
					0.457382253	0.56725964	1.240230981	
					0.344656315	BD	BD	
					0.213661001	0.71407978	3.342115673	
					0.312263805	0.0173547	0.055577046	
1.8	Marine	Superior province of the Canadian Shield		Black shale	0.075145595	8.00837E-05	0.000841353	2
					0.09518442	0.000101439	0.00120887	
					0.083912581	8.94268E-05	0.002163722	
					0.041330077	4.4046E-05	0.000799286	
					0.055106769	5.87281E-05	0.000625219	
					0.093931993	0.000100105	0.001310304	
					0.076398021	8.14184E-05	0.000305204	
					0.266766861	0.000284297	0.000804954	
					0.353184295	0.000376393	0.001502657	
					0.250485315	0.000266946	0.001065714	
1.65					0.046145582	8.72245E-05	0.001890203	This study

1.4	Marine	Sedimentary	Cuddapah Supergroup, Cumbum Formation, India	Black shale	0.052413282	0.000120526	0.002299531	10
1.72	Deep sea marine sediments	Sedimentary (seafloor-hydrothermal Si-Fe-Mn exhalites)	Pecos greenstone belt, New Mexico, USA	Ferruginous/ manganous formation	0.666290939	3.33682E-05	5.00805E-05	11
					0.275784332	5.33891E-06	1.9359E-05	
					0.276285303	3.33682E-05	0.000120774	
					1.132569353	9.74352E-05	8.60302E-05	
					0.751080218	5.60586E-05	7.46373E-05	
					0.322124115	5.60586E-05	0.000174028	
					0.546934686	3.06988E-05	5.61287E-05	
					0.320621204	2.80293E-05	8.74219E-05	
					0.236458138	0.000158833	0.000671716	
					0.028055771	4.39126E-05	0.001565188	
					0.045399339	0.000166708	0.003672027	
					0.025760299	5.47239E-05	0.002124349	
					0.022699669	7.54122E-05	0.00332217	
					0.021041828	7.3944E-05	0.003514142	
					0.10763214	0.000101306	0.000941223	
					0.061467644	0.000304185	0.004948695	
					0.140023803	9.07615E-06	6.48187E-05	
					0.104954089	6.39335E-05	0.000609157	
					0.052030703	0.000299513	0.005756468	
					0.047949863	0.000258537	0.005391818	
					0.053688544	0.000362379	0.006749649	
1.45	Fluvial - marine (Libby, Bonner, McNamara & Mount Shields formation)	Sedimentary	Belt-Purcell sequence, Western North America	Fine-grained siliciclastic/subordinate coarse sandstones	0.072765984	0.00014949	0.002054388	12
					0.105454318	0.000140547	0.001332775	
					0.083912581	BD	BD	
					0.039326195	6.13975E-06	0.000156124	
					0.066754337	1.48155E-05	0.00022194	
					0.035193187	BD	BD	
					0.008641743	1.12117E-05	0.001297391	
					0.02354562	8.27532E-06	0.000351459	
					0.021291252	1.01439E-05	0.000476437	
					0.045838813	5.73933E-05	0.001252068	
					0.042832989	9.3431E-06	0.000218129	
					0.051474732	9.20963E-06	0.000178915	
					0.112092179	0.0002229	0.001988539	
					0.108961112	8.71578E-05	0.000799898	
					0.048594151	BD	BD	
					0.049345607	1.85527E-05	0.000375975	
					0.068257248	2.72285E-05	0.000398909	
					0.03018348	4.83172E-05	0.001600782	
					0.001377669	BD	BD	

					0.006637861	3.09657E-05	0.004665012	
					0.006763104	1.06778E-05	0.001578836	
					0.064374726	8.26197E-05	0.001283418	
					0.057862108	4.15101E-05	0.000717396	
					0.047341725	3.13661E-05	0.000662547	
					0.050973762	3.45695E-05	0.000678182	
					0.036570856	3.53703E-05	0.000967172	
					0.047216482	8.36875E-05	0.001772421	
					0.037322312	4.76498E-05	0.001276711	
					0.085039765	1.13452E-05	0.00013341	
					0.036946584	0.000108647	0.002940648	
					0.019913583	0.000638267	0.032051852	
					0.051099004	8.15519E-05	0.001595959	
					0.036320371	2.26904E-05	0.000624729	
					0.057862108	1.42816E-05	0.000246821	
					0.034441731	7.76812E-05	0.002255438	
					0.02354562	1.12117E-05	0.00047617	
					0.048093181	BD	BD	
					0.034817459	1.44151E-05	0.000414018	
					0.051725218	3.10992E-05	0.000601238	
					0.052977644	0.000131604	0.002484147	
					0.039075709	3.49699E-05	0.000894927	
					0.055858225	2.30908E-05	0.000413382	
					0.054355313	7.20753E-06	0.0001326	
					0.109086355	0.000274821	0.002519294	
					0.026676686	BD	BD	
					0.040829106	1.16121E-05	0.000284408	
					0.020665039	8.80921E-06	0.000426286	
					0.04333396	1.29469E-05	0.00029877	
					0.073266955	1.29469E-05	0.000176708	
					0.038950467	1.80188E-05	0.000462609	
					0.054731041	1.58833E-05	0.000290206	
					0.039952408	1.45485E-05	0.000364147	
					0.058363078	2.60272E-05	0.000445953	
					0.032813576	9.36979E-05	0.002855463	
					0.047466967	8.99607E-05	0.001895228	
					0.049220364	1.36142E-05	0.000276598	
					0.031686392	0.000127333	0.004018542	
					0.037197069	1.02774E-05	0.000276296	
					0.0358194	BD	BD	
					0.026175715	1.56163E-05	0.000596596	
					0.008140773	1.32138E-05	0.001623164	
					0.075270837	2.29573E-05	0.000304996	
					0.025173774	1.56163E-05	0.000620341	
					0.031185422	8.54226E-06	0.000273918	

					0.046339783	4.67155E-06	0.000100811	
					0.048093181	2.10887E-05	0.000438497	
					0.060116476	BD	BD	
					0.018410671	1.36142E-05	0.000739475	
					0.042707746	7.34101E-06	0.000171889	
					0.062370844	2.46925E-05	0.000395898	
					0.062245601	8.40879E-06	0.000135091	
					0.054605799	1.41481E-05	0.000259096	
					0.034566974	7.20753E-06	0.000208509	
					0.066128123	2.57603E-05	0.000389551	
					0.063498027	9.30306E-05	0.001465094	
~0.83	Shallow submarine rifted continental margin	Sedimentary	Shilu Fe-Co-Cu ore district, Western Hainan Province, South China	Siliceous BIFs	0.912832772	0.000136142	0.000149143	13
					1.046607789	7.63854E-05	7.29838E-05	
					1.02084749	0.000226904	0.00022227	
					1.119297742	6.74038E-05	6.02197E-05	
					0.890260628	4.67155E-05	5.2474E-05	
					0.88885784	4.33787E-05	4.88027E-05	
					0.697823544	0.000162837	0.00023335	
					0.29178002	0.00011145	0.000381965	
					0.39061285	BD	BD	
					0.662371251	0.000331013	0.000499739	
					0.403748052	BD	BD	
					0.79104522	0.000173515	0.000219349	
					0.471974586	0.00056459	0.00119623	
					0.429508351	7.54122E-05	0.000175578	
~0.75	Marine	Sedimentary	Urucum district, Mato Grosso do Sul, Brazil	BIF/Mn Formation	0.880313582	4.80502E-05	5.45831E-05	14
					0.689210345	2.9364E-05	4.26053E-05	
					0.392143165	2.80293E-05	7.14772E-05	
					0.934257179	9.07615E-05	9.71483E-05	
					0.771278654	7.34101E-05	9.51797E-05	
					0.659948252	0.000112117	0.000169888	
					0.188740686	2.53598E-05	0.000134363	
					0.100319369	3.06988E-05	0.00030601	
					0.159058175	0.00012413	0.000780405	
0.716	Marine	Sedimentary	Rapitan, Northern Canadian Cordillera	BIFs, Hematite-rich	0.021114049	2.00209E-05	0.000948228	15
					1.061172395	1.33473E-05	1.25779E-05	
					0.999179506	0.000204213	0.000204381	
					6.98331662	9.07615E-05	1.29969E-05	
					2.242683927	0.000125464	5.59439E-05	
					2.206217522	0.000136142	6.17085E-05	
					3.136110858	0.00056459	0.000180029	
					3.519008114	0.000117456	3.33776E-05	
					5.050597137	9.87699E-05	1.95561E-05	
					17.95970462	7.20753E-05	4.01317E-06	
					6.108122892	8.54226E-05	1.39851E-05	

					2.461482359	5.07197E-05	2.06053E-05	
					4.028990792	5.07197E-05	1.25887E-05	
					5.170024615	4.00419E-05	7.745E-06	
0.54	Marine	Sedimentary Shallow continental shelf	Northern Tarim Basin, Northwest China	Non-pyritic Black shales	0.030351243	0.000527218	0.01737055	16
					0.039660658	0.002215649	0.05586517	
					0.031754032	0.000545904	0.017191643	
					0.072307374	0.000623318	0.008620397	
					0.093349202	0.000595289	0.006377012	
					0.076260687	0.000425778	0.005583197	
					0.056239068	0.000373724	0.006645274	
					0.050117809	0.000316331	0.006311742	
0.54	Marine	Southern Sweden		Pyritic black shale	0.097243369	0.002669457	0.027451302	17
					0.097243369	0.004004186	0.041176954	
					0.097243369	0.000308322	0.003170625	
					0.097243369	1.06778E-05	0.000109805	
					0.097243369	9.3431E-06	9.60796E-05	
					0.097243369	0.013347286	0.137256512	
					0.097243369	0.002669457	0.027451302	
					0.097243369	0.000154829	0.001592176	
					0.097243369	6.67364E-05	0.000686283	
					0.097243369	0.000157498	0.001619627	
					0.090093121	0.004004186	0.044444966	
					0.090093121	0.003336821	0.037037472	
					0.090093121	0.000428448	0.004755611	
					0.090093121	2.66946E-05	0.0002963	
					0.090093121	3.33682E-05	0.000370375	
					0.091691412	0.004004186	0.043670237	
					0.091691412	0.001334729	0.014556746	
					0.091691412	0.000436456	0.004760056	
					0.091691412	4.00419E-05	0.000436702	
					0.091691412	6.00628E-05	0.000655054	
					0.086980661	0.005338914	0.061380475	
					0.086980661	0.000667364	0.007672559	
					0.086980661	0.000552578	0.006352879	
					0.086980661	0.000106778	0.00122761	
					0.086980661	0.000617979	0.00710479	
					0.090093121	0.006673643	0.074074943	
					0.090093121	0.002002093	0.022222483	
					0.090093121	0.000715415	0.007940834	
					0.090093121	4.4046E-05	0.000488895	
					0.090093121	6.94059E-05	0.000770379	
					0.088578951	0.004004186	0.045204709	
					0.088578951	0.000667364	0.007534118	
					0.088578951	0.000421774	0.004761563	
					0.088578951	8.00837E-05	0.000904094	

					0.088578951	0.000217561	0.002456123	
					0.088578951	0.006673643	0.075341181	
					0.088578951	0.001334729	0.015068236	
					0.088578951	0.000703402	0.007940961	
					0.088578951	6.67364E-05	0.000753412	
					0.088578951	0.000141481	0.001597233	
					0.094887993	0.0093431	0.098464512	
					0.094887993	0.002002093	0.021099538	
					0.094887993	0.001054436	0.011112424	
					0.094887993	6.27322E-05	0.000661119	
					0.094887993	0.000132138	0.00139257	
					0.08538237	0.003336821	0.039080918	
					0.08538237	0.000667364	0.007816184	
					0.08538237	0.000339021	0.003970621	
					0.08538237	6.67364E-05	0.000781618	
					0.08538237	0.000136142	0.001594501	
					0.094887993	0.002669457	0.028132718	
					0.094887993	0.001334729	0.014066359	
					0.094887993	0.000301649	0.003178997	
					0.094887993	2.66946E-05	0.000281327	
					0.094887993	3.47029E-05	0.000365725	
					0.099598745	0.002002093	0.020101587	
					0.099598745	0.000654017	0.006566518	
					0.099598745	0.000237582	0.002385388	
					0.099598745	2.66946E-05	0.000268021	
					0.11230095	0.004004186	0.035655849	
					0.11230095	0.001334729	0.011885283	
					0.11230095	0.000535226	0.004765998	
					0.11230095	4.00419E-05	0.000356558	
					0.11230095	6.67364E-05	0.000594264	
					0.105991908	0.004004186	0.037778221	
					0.105991908	0.001334729	0.01259274	
					0.105991908	0.000504527	0.004760056	
					0.105991908	4.00419E-05	0.000377782	
					0.105991908	6.4067E-05	0.000604452	
0.54	Marine	Sedimentary	Niutitang formation, Guizou Province, China	Sulphidic/non-sulphidic black shales with soft bodied fossils	0.016009018	0.000540565	0.033766285	18
					0.103743481	0.00137477	0.013251632	
					0.073364163	0.001454854	0.019830583	
					0.040085573	0.004778328	0.119203192	
					0.032144092	0.005392303	0.167754107	
					0.004916155	0.000654017	0.133034245	
					0.196772264	0.182590868	0.927929905	
					0.093028783	0.008875945	0.095410739	
					0.083574639	0.00265611	0.03178129	
					0.07235572	0.001881967	0.026009931	

					0.073364163	0.001908662	0.02601627	
					0.004916155	4.47134E-05	0.009095198	
					0.033278589	9.04946E-05	0.002719304	
					0.014244245	2.72285E-05	0.001911541	
					0.056346702	0.00016297	0.002892279	
					0.19021739	0.003630462	0.019085856	
					0.054203763	0.000667364	0.012312139	
					0.004411934	9.47657E-05	0.021479407	
0.52	Marine (shallow continental shelf to deepsea)	Sedimentary	Chengjiang, South China		0.052601916	-2.00209E-05	-0.000380612	This study
					0.076773749	0.000232243	0.003025028	
					0.078902874	0.000204213	0.002588163	
					0.080781514	0.000164172	0.002032292	
					0.071513558	8.54226E-05	0.001194496	
					0.068758219	8.54226E-05	0.001242362	
					0.07777569	0.000133473	0.001716126	
					0.083912581	0.000157498	0.001876929	
					0.074018411	0.000238916	0.003227797	
					0.076523264	0.000186862	0.002441898	
					0.075145595	0.00012146	0.001616333	
					0.076147536	0.000160167	0.002103383	
					0.075270837	5.60586E-05	0.000744759	
					0.094808692	0.000353703	0.003730703	
					0.076022293	0.000309657	0.00407324	
					0.079028117	0.000138812	0.001756486	
					0.073392197	0.000319	0.004346513	
					0.073141712	0.000226904	0.00310225	
					0.072014528	0.000308322	0.00428139	
					0.069509675	0.000138812	0.001997014	
					0.069885403	0.000182858	0.002616538	
					0.080531029	0.000228239	0.002834169	
					0.083286367	0.000132138	0.001586552	
					0.083161125	0.000306988	0.003691479	
					0.077525205	0.000244255	0.003150657	
					0.071513558	0.000256268	0.003583487	
					0.083161125	0.000229573	0.002760585	
					0.086667919	0.000126799	0.001463047	
					0.077024234	0.000262942	0.003413751	
					0.068883462	0.000198875	0.002887116	

					0.072765984	0.000213557	0.002934841	
					0.068632976	0.000475163	0.006923252	
					0.070511616	0.000339021	0.004808017	
					0.071263072	0.000302983	0.004251618	
					0.070636859	0.000202879	0.002872137	
					0.065627153	0.000285632	0.004352344	
					0.071889285	0.000184193	0.00256217	
					0.075646565	0.000419105	0.005540301	
					0.075020352	0.000297644	0.003967516	
					0.071764043	0.000266946	0.00371977	
					0.075145595	0.000153494	0.002042619	
					0.074394139	0.000178854	0.002404136	
					0.078151418	0.000208218	0.002664285	
					0.074268896	0.000320335	0.004313176	
					0.080155301	0.000237582	0.002964017	
					0.074394139	0.000323004	0.004341798	
					0.070010646	0.000391075	0.005585943	
					0.071012587	0.000230908	0.003251649	
					0.072891227	0.000173515	0.002380461	
					0.0716388	0.000230908	0.003223226	
					0.068382491	0.000201544	0.002947304	
					0.07965433	0.000365716	0.004591284	
					0.067756278	0.000188197	0.002777554	
					0.073016469	0.000241586	0.003308649	
					0.077650448	0.000340356	0.004383179	
					0.078401904	0.000343025	0.004375216	
					0.078777632	0.000456477	0.005794502	
					0.076272779	0.000301649	0.003954867	
					0.076147536	0.000210887	0.002769454	
					0.070135888	0.000277624	0.003958366	
					0.073893168	0.000391075	0.005292444	
					0.081908698	0.000383067	0.004676757	
					0.081908698	0.00024826	0.00303093	
					0.084538794	0.000264276	0.003126094	
					0.080030058	0.000361711	0.004519695	
					0.075521323	0.000545904	0.007228475	
					0.065877638	0.000101439	0.001539815	

					0.074895109	0.000112117	0.00149699	
					0.064124241	0.000726092	0.011323212	
					0.071263072	0.00066603	0.009346069	
					0.070511616	0.000424444	0.006019486	
					0.074143653	0.000389741	0.005256562	
					0.060241718	0.000253598	0.004209681	
					0.05560774	0.000319	0.005736614	
					0.061118417	0.000192201	0.00314473	
					0.062997057	0.00046582	0.007394318	
					0.036445613	-1.73515E-05	-0.000476092	
					0.042958232	1.4682E-05	0.000341774	
					0.04947085	3.73724E-05	0.000755443	
					0.050598034	-5.73933E-05	-0.0011343	
					0.04571357	0.000134808	0.002948962	
					0.046339783	1.20126E-05	0.000259228	
					0.047216482	-8.00837E-06	-0.00016961	
					0.046965997	-3.73724E-05	-0.000795733	
					0.05134949	-1.20126E-05	-0.000233937	
					0.048594151	1.33473E-06	2.74669E-05	
					0.053979585	2.53598E-05	0.000469804	
					0.056734924	0.000137477	0.002423147	
					0.046715511	2.66946E-06	5.71428E-05	
					0.04947085	-7.20753E-05	-0.001456926	
					0.052727159	2.66946E-06	5.06277E-05	
					0.052977644	0.000117456	0.002217088	
					0.057110652	3.60377E-05	0.000631015	
					0.052476674	1.86862E-05	0.000356086	
					0.05986599	8.40879E-05	0.001404602	
					0.054605799	0.000152159	0.0027865	
					0.05936502	8.40879E-05	0.001416455	
					0.061995116	0.000101439	0.001636248	
					0.055983468	4.53808E-05	0.00081061	
					0.055357255	7.07406E-05	0.001277892	
					0.059490262	3.60377E-05	0.000605774	
					0.05748638	8.94268E-05	0.001555617	

					0.060742689	3.60377E-05	0.000593284	
					0.050472791	9.07615E-05	0.001798227	
					0.046965997	4.53808E-05	0.000966247	
					0.060742689	0.000144151	0.002373136	
					0.05798735	9.74352E-05	0.001680283	
					0.065376667	7.20753E-05	0.001102463	
					0.055732983	0.000284297	0.005101058	
					0.055858225	4.9385E-05	0.000884113	
					0.056359196	0.000154829	0.002747174	
					0.052351431	0.000128134	0.002447573	
0.48	Marine	Sedimentary	Canadian Appalachian, Canada	Black shale	0.058989292	4.27113E-05	0.000724052	19
					0.056359196	7.20753E-05	0.001278857	
					0.025674745	0.001121172	0.043668282	
					0.01553009	0.000345695	0.022259672	
					0.114221304	0.000246925	0.00216181	
					0.061995116	0.000880921	0.01420952	
					0.066002881	0.000213557	0.003235564	
					0.088170831	0.000113452	0.001286729	
0.093	Marine	Sedimentary	Atlantic Ocean ocean drilling project	Black shale	3.426535231	0.000173515	5.06385E-05	20, 21
					4.592852001	0.000333682	7.26525E-05	
					4.640290626	0.000280293	6.04042E-05	
					5.438116035	0.000240251	4.41791E-05	
					3.497117378	0.000333682	9.54163E-05	
					4.318982183	0.000213557	4.9446E-05	
					5.09744525	0.000133473	2.61843E-05	
0.072	Marine	Sedimentary	Atlantic Ocean	Black shale	0.400776505	0.000427113	0.001065714	21
0.002	Marine	Sedimentary	Milos Island, Greece	Hydrothermally-derived hematic IF	0.096937817	0.018085572	0.186568799	22
					0.190869811	0.00954064	0.049985064	
					0.232575615	0.010600414	0.045578357	
					0.217546497	0.109567868	0.503652644	
					0.51787839	0.029243903	0.056468668	
~0	Marine	Sedimentary	Atlantic Ocean	Black shale	0.134009644	0.000961005	0.00717116	21
					0.162815455	0.000173515	0.001065714	

DB=below detection limit. IF= Iron Formation. BIF=Banded Iron Formation. BIF, Banded Iron Formation. IF, Iron Formation.

Supplementary Table 2. Analysis of variance involving multiple comparisons between the six key sedimentary arsenic stages identified in this study. The independent variable is the log As/Fe molar ratio.

(I) Stage	(J) Stage	Mean Difference (I-J)	Std. Error	Sig.	95% Confidence Interval	
					Lower Bound	Upper Bound
Stage I	Stage II	-,68578	,37916	,071	-1,4312	,0597
	Stage III	-,33560	,38677	,386	-1,0960	,4248
	Stage IV	-1,51048*	,36963	,000	-2,2372	-,7837
	Stage V	-,46214	,38809	,234	-1,2252	,3009
	Stage VI	-2,26008*	,37015	,000	-2,9878	-1,5323
Stage II	Stage I	,68578	,37916	,071	-,0597	1,4312
	Stage III	,35018*	,17260	,043	,0108	,6895
	Stage IV	-,82470*	,12971	,000	-1,0797	-,5697
	Stage V	,22364	,17552	,203	-,1214	,5687
	Stage VI	-1,57431*	,13119	,000	-1,8322	-1,3164
Stage III	Stage I	,33560	,38677	,386	-,4248	1,0960
	Stage II	-,35018*	,17260	,043	-,6895	-,0108
	Stage IV	-1,17488*	,15051	,000	-1,4708	-,8790
	Stage V	-,12654	,19140	,509	-,5029	,2498
	Stage VI	-1,92448*	,15178	,000	-2,2229	-1,6261
Stage IV	Stage I	1,51048*	,36963	,000	,7837	2,2372
	Stage II	,82470*	,12971	,000	,5697	1,0797
	Stage III	1,17488*	,15051	,000	,8790	1,4708
	Stage V	1,04834*	,15386	,000	,7459	1,3508
	Stage VI	-,74960*	,10037	,000	-,9469	-,5523
Stage V	Stage I	,46214	,38809	,234	-,3009	1,2252
	Stage II	-,22364	,17552	,203	-,5687	,1214
	Stage III	,12654	,19140	,509	-,2498	,5029
	Stage IV	-1,04834*	,15386	,000	-1,3508	-,7459
	Stage VI	-1,79794*	,15510	,000	-2,1029	-1,4930
Stage VI	Stage I	2,26008*	,37015	,000	1,5323	2,9878
	Stage II	1,57431*	,13119	,000	1,3164	1,8322
	Stage III	1,92448*	,15178	,000	1,6261	2,2229
	Stage IV	,74960*	,10037	,000	,5523	,9469
	Stage V	1,79794*	,15510	,000	1,4930	2,1029

*. The mean difference is significant at the 0.05 level.

Supplementary Table 3. Analysis of variance within stage IV. The independent variable is the log As/Fe molar ratio.

	Sum of Squares	df	Mean Square	F	Sig.
Between Groups	60,594	7	8,656	29,721	,000
Within Groups	37,281	128	,291		
Total	97,875	135			

Supplementary references

1. Dymek, R. F. & Klein, C. Chemistry, petrology and origin of banded iron-formation lithologies from the 3800 MA Isua supracrustal belt, West Greenland. *Pre. Res.* **39**, 247-302 (1988).
2. Cameron, E. M. & Garrels, R. M. Geochemical compositions of some Precambrian shales from the Canadian Shield. *Chem. Geol.* **28,** 181–197 (1980).
3. Requia, K. & Fontboté, L. In Mineral deposits: processes to processing (eds C.J. Stanley et al. 1999) p. 1025-1028 (Balkema, Amsterdam).
4. Manikyamba, C. & Kerrich, R. Geochemistry of black shales from the Neoarchaean Sandur Superterrane, India: First cycle volcanogenic sedimentary rocks in an intraoceanic arc–trench complex. *Geochim. Cosmo. Acta* **70,** 4663–4679 (2006).
5. Maboko, M. The geochemistry of banded iron formations in the sukumaland greenstone belt of Geita, northern Tanzania: evidence for mixing of hydrothermal and clastic sources of the chemical elements. *Tan. J. Sci. 27, 21–36 (2004).*
6. Percoits, E. *et al*. Petrography and geochemistry of the Dales Gorge banded iron formation: Paragenetic sequence, source and implications for palaeo-ocean chemistry. *Pre. Res.* **172**, 163–187 (2009).
7. Spier, C. A. Sonia De Oliveira, M. B. Alcides, N. & Rios, F. J. Geochemistry and genesis of the banded iron formations of the Cauê Formation, Quadrilátero Ferrífero, Minas Gerais, Brazil. *Pre. Res.* **152,** 170–206 (2007).
8. Beukes, N. J. & Klein, C. Geochemistry and sedimentology of a facies transition — from microbanded to granular iron-formation — in the early Proterozoic Transvaal Supergroup, South Africa. *Precam. Res.* **47,** 99–139 (1990).
9. Caddey, S. W. Bachman, R. L. Campbell, T. J. Reid, R. R. & Otto, R. P. The Homestake gold mine, an Early Proterozoic iron-formation hosted gold deposit Lawrence County South Dakota. *U.S. Geol. Sur. Bull.* **1857,** J1–J67 (1991).
10. Manikyamba, C. Kerrich, R. González-Álvarez, I. Mathur, R. & Khanna, C. T. Geochemistry of Paleoproterozoic black shales from the Intracontinental Cuddapah basin, India: implications for provenance, tectonic setting, and weathering intensity*Pre. Res.* **162,** 424–440 (2008).
11. Slack, J. F. Tor, G. & Bekker, A. Seafloor-hydrothermal Si-Fe-Mn exhalites in the Pecos greenstone belt, New Mexico, and the redox state of ca. 1720 Ma deep seawater. *Geosphere* **5**, 302–3014 (2009).
12. González-Álvarez, I. Geochemical study of the Mesoproterozoic Belt-Purcell Supergroup, Western North America: Implications for provenance, weathering and diagenesis. PhD Thesis, University of Saskatchewan Saskatoon (2005).
13. Xu, D. R. *et al*. Petrography and geochemistry of the Shilu Fe–Co–Cu ore district, South China: Implications for the origin of a Neoproterozoic BIF system. *Ore Geol. Rev.* **57,** 322–350 (2013).
14. Klein, C. & Ladeira, E. Geochemistry and mineralogy of Neoproterozoic banded iron formations and some selected, siliceous manganese formations from the Urucum District, Mato Grosso do Sul, Brazil. *Econ. Geol.* **99,** 1233–1244 (2004).

15. Halverson, G. P. *et al*. Fe isotope and trace element geochemistry of the Neoproterozoic syn-glacial Rapitan iron formation. *Earth Plan. Sci. Lett.* **309,** 100–112 (2011).
16. Yu, B. Dong, H. Widom, E. Chen, J. Lin, C. Geochemistry of basal Cambrian black shales and cherts from the Northern Tarim Basin, Northwest China: Implications for depositional setting and tectonic history. *J. Asian Earth Sci.* **34,** 418–636 (2009).
17. Armands, G. Geochemical studies of uranium, molybdenum, and vanadium in Swedish Alum shale. Acta Universitatas Stockholmiensis. Stockholm contributions in geology VOL. XXVII:1. PhD thesis, http://su.diva-portal.org/smash/record.jsf?pid=diva2:564876 (1972).
18. Pašav, J., Kríbek, B. Vymazalová, A., Sýkrorová, I., Žák, K. & Orberger, B. Multiple Sources of Metals of Mineralization in Lower Cambrian Black Shales of South China: Evidence from Geochemical and Petrographic Study. *Res. Geol.* **58,** 25–42 (2007).
19. Fyffe, L. R. & Pickerill, R. K. Geochemistry of Upper Cambrian-Lower Ordovician black shale along a northeastern Appalachian transect. *Geol. Soc. Am. Bull.* 7**,** 897–910 (2013).
20. Dean, W. E. Arthur, M. A. Stow, D. A. V. Origin and geochemistry of cretaceous deep-sea black shales and multicolored claystones, with emphasis on deep sea drilling project site 530, southern Angola basin. *Proj. Rep. Pub.* doi:10.2973/dsdp.proc.75.121.1984 (2007).
21. von Frau Almut Bunte Geochemical signatures of black shales deposited during Oceanic Anoxic Event 2 (Cenomanian/Turonian) in the tropical Atlantic (Demerara Rise, ODP Leg 207) and in northern Germany (Wunstorf). PhD Thesis, University. Von der Fakultät für Mathematik und Naturwissenschaften der Carl von Ossietzk Universität. PhD Thesis (2009). http://www.mbgc.icbm.de/download/Bunte_Diss09_Abstract.pdf.
22. Chi Fru, E. *et al*. Fossilized iron bacteria reveal a pathway to the biological origin of banded iron formation. *Nat. Comm.* **4**, 2050 doi:10.1038/ncomms3050 (2013).
